# Transduction of the rat brain by Bovine Herpesvirus 4

**DOI:** 10.1186/1479-0556-6-6

**Published:** 2008-02-12

**Authors:** Marco Redaelli, Andrea Cavaggioni, Carla Mucignat-Caretta, Sandro Cavirani, Antonio Caretta, Gaetano Donofrio

**Affiliations:** 1 Department of Human Anatomy and Physiology, University of Padova, 35131 Padova, Italy; 2 Department of Neuroscience, University of Padova, 35131 Padova, Italy; 3 Department of Animal Health, University of Parma, 43100 Parma, Italy; 4 Department of Pharmaceutical Sciences, University of Parma, 43100 Parma, Italy

## Abstract

Bovine herpesvirus 4 (BoHV-4) is a gamma-herpesvirus with no clear disease association. A recombinant BoHV-4 (BoHV-4EGFPΔTK) expressing Green Fluorescent Protein (EGFP), was successfully used to infect F98 rat glioma cells. BoHV-4EGFPΔTK was injected into the lateral ventricle of the rat brain. Histology and immunohistochemistry showed that ependymal and rostral migratory stream cells were transduced while neurons were not. Clinical scores, evaluated for 90 days, indicated that the virus was non neuropathogenic, suggesting this virus is a suitable vector for brain tumor gene therapy.

## Text

Gene delivery and targeting is a major issue in the treatment of severe brain tumors. The cancer treatment mediated or coadiuvated by genetically modified oncolytic viruses is an interesting opportunity in clinical oncology.

Bovine herpesvirus 4 (BoHV-4) is a gamma-herpesvirus with no clear disease association [1], suggesting it as a suitable vector for gene therapy. BoHV-4 has been isolated from different tissues and has been show to establish a persistent infection in its natural host, the cattle, and in the experimental animal, the rabbit [2,3]. In the natural and experimental host some evidence indicates that the monocyte/macrophage lineage is a site of persistent infection [4,5]. Interestingly, unlike other gamma-herpes viruses like Epstein-Barr Virus [6] and Herpes Virus Saimiri [7], BoHV-4 is non oncogenic. Hence, BoHV-4 could be employed as a possible therapeutic candidate as attenuation of genes to render it non-pathogenic is not required. However, BoHV-4 does replicate and cause a cytopathic effect in a number of immortalized cell lines and primary cell cultures [8,9].

We have previously demonstrated that BoHV-4 does not replicate in the mouse brain and that infection was restricted to ependymal and rostral migratory stream (RMS) regions after viral injection in the lateral ventricle of the mouse brain [10]. The aim of this work was to evaluate the suitability of BoHV-4 as a vector for glioma gene therapy. The virus was first assessed *in vitro*, using the rat glioma F98 cell line (ATCC, USA) and then *in vivo *by injecting the virus into the brain of rats.

**Figure 1:**
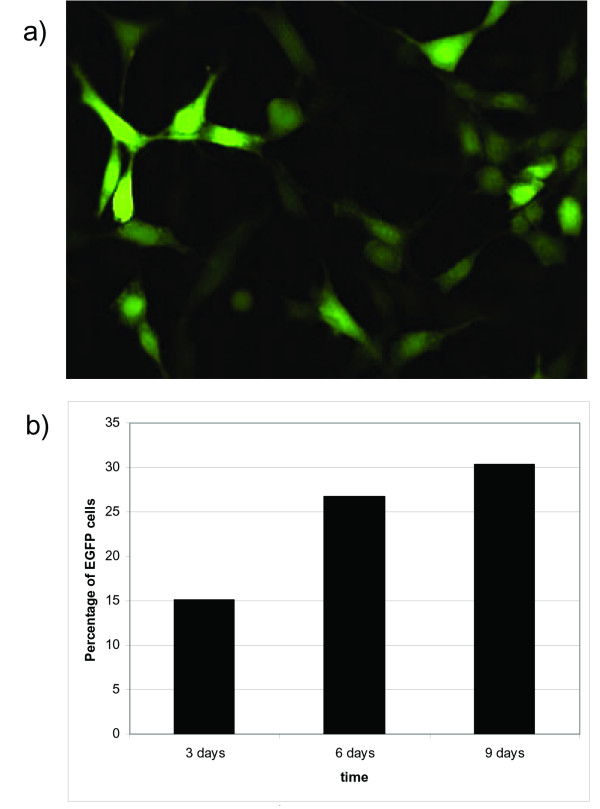
**Rat glioma F98 cells culture infected with BoHV-4EGFPΔTK. (a), epifluorescence; (b) Representative image of BoHV-4EGFPΔTK infected F98 cells expressing EGFP**.

The infection and transduction of rat glioma cells *in vitro *was explored, employing the rat glioma F98 cell line, which were maintained in growth medium (90% DMEM, 10% FBS, 100 IU/ml penicillin, 10 μg/ml streptomycin), at 37°C in a humidified incubator with 95% air and 5% CO_2_. F98 cells were cultured till 80–90% confluent (4–6 days) and exposed to a multiplicity of infection (m.o.i.) of 5 with recombinant BoHV-4 (BoHV-4EGFPΔTK) obtained by the insertion of an EGFP gene into the TK locus of the BoHV-4 genome [8], allowing rapid monitoring of the cell infection through EGFP expression. Cells were monitored for 9 days with an epiflorescence microscope and the establishment of infection was detectable as early as 48 h post-infection (Fig 1a and 1b). Cells were successfully transduced with an efficiency ranging from ~15% after 3 days to ~30% after 9 days post infection, despite the medium being changed every 3 days. Cytopathic effects (CPE) were observed following infection.

*In vitro*, BoHV-4 is able to replicate in primary cell culture or cell lines from a broad spectrum of host species. The infection in some permissive cells leads to viral progeny and CPE; in other cells, although CPE takes place, no viral progeny is produced; whereas in some non permissive cells BoHV-4 infection is persistent with no effect on cell survival [11]. The nature of cell death induced by BoHV-4 is highly controversial. For some cell types, it is mediated by apoptosis [12-14], but in other cells BoHV-4 infection protects against TNF-alpha induced apoptosis [12]. Since BoHV-4 induced CPE in F98 cells, the nature of cell death was investigated. F98 cells were infected with 5 m.o.i. of BoHV-4 and cell death was examined by Wright's nuclear staining with propidium iodide and by internucleosomal DNA fragmentation. Both approaches showed that BoHV-4-induced CPE was not mediated by apoptosis (data not shown).

**Figure 2:**
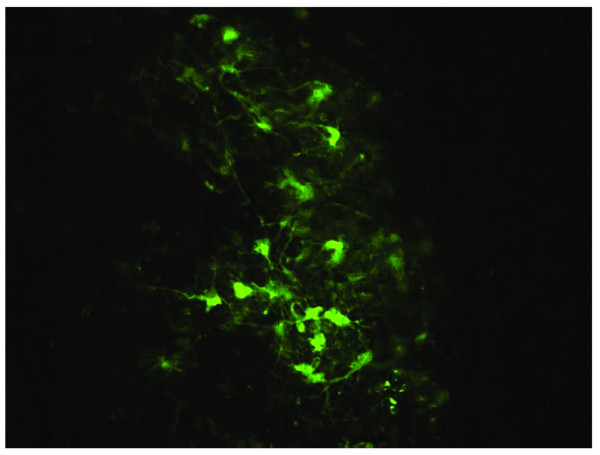
Rat RMS area 6 days post BoHV-4EGFPΔTK injection. EGFP expression, 20× epifluorescence.

With the assumption that BoHV-4 had a replicating competent behavior in F98 cells that led to CPE, the outcome of infection following BoHV-4 inoculation into the adult rat brains was investigated to rule out a possible neuropathogenic effect. All animals were cared for and used in accordance with the Italian laws for animal experimentation. Wistar rats were maintained at 24°C with a controlled light cycle (12 h light, starting from 06:00 a.m.) and with food and water ad libitum. For intracerebral virus injection, 21 four-month-old male Wistar rats (3 rats per group and per time point) were pre-anaesthetized with isoflurane and then anaesthetized with ketamine (20 mg/kg body weight) and xylazine (75 mg/kg body weight). Rats were inoculated with a high dose (12 × 10^6 ^Tissue Infectious Dose 50, TCID50, corresponding to 12 μl) of BoHV-4EGFPΔTK into the left lateral ventricle of the brain, by a Hamilton syringe (0.5 μl/min) using the stereo tactic coordinates from bregma (AP +1, ML -1.5, DV -3.7 mm). Throughout the experiment, each animal was monitored daily to determine the degree of clinical impairment until 90 days post inoculation (p.i.), using a visual assessment scale [10]. Interestingly, all rats inoculated with the virus showed no clinical signs. The transduction capability of BoHV-4EGFPΔTK was analyzed through EGFP expression in serial rat brain section, at 4, 6, 14, 27, 45, 60, and 90 days p.i.. Anaesthetized rats were perfused with PBS for 15 min and then with 4% formalin in PBS for 30 min. Brains were carefully removed, post-fixed for 2 hours at 4°C (with 4% formalin in PBS), equilibrated for 24 h in 30% sucrose/PBS at 4°C and frozen at -80°C until cryostat sectioning. Brain sections were stored at -20°C. After thawing, brains sections were observed with an epifluorescence microscope. EGFP labelled cells were mapped using the Paxinos and Watson atlas [15] and EGFP expression was observed as early as 4 days p.i., till 60 days p.i. (see representative image, Fig. 2) and mainly localized in two areas: in the proximity of the lateral ventricle border and in the Rostral Migratory Stream (RMS). The percentage of transduction was ~10% up to day 60 p.i., however at day 90 p.i. the EGFP signal disappeared (percentage of transduction was calculated on the basis of 10 slices for each brain and 5 fields of view for each slice. The ratio between EGFP positive cells on DAPI counterstained blue cells was made. Data were expressed as ± SEM. Statistical significance of differences was determined by the unpaired student's *t *test. Differences at P < 0.05 were considered to be statistically significant).

**Figure 3:**
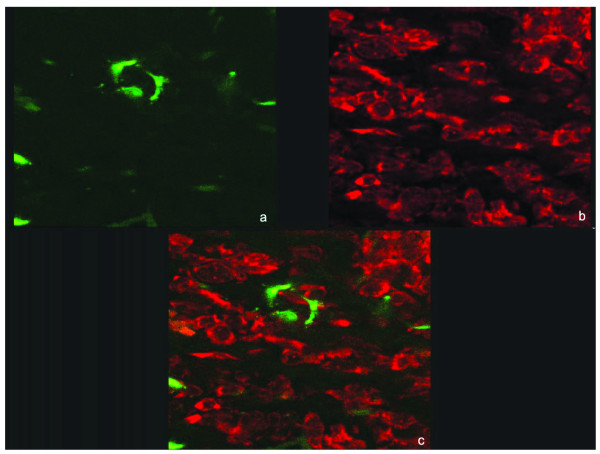
Rat RMS 96 hours post BoHV-4EGFPΔTK injection, EGFP expression (a), NeuroTrace™ staining in red (b), merge (c), 40× oil confocal.

**Figure 4:**
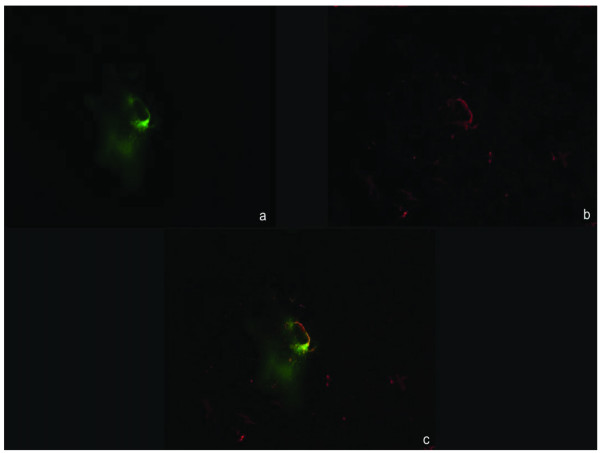
Rat RMS 6 days post BoHV-4EGFPΔTK injection, EGFP expression (a), GFAP immunostainig (b), co-localization (c), 20× epifluorescence.

Because the EGFP signal was localised to the area of inoculum and did not invade the parenchyma and cause clinical signs, this indicated that BoHV-4EGFPΔTK infection was unpermissive in the rat brain, as compared to the replication-competent behaviour of BoHV-4EGFPΔTK observed in glioma cells *in vitro*. To characterize further EGFP expressing cells in the transduced rat brains, Neurotrace stain (Molecular Probes) was used according to the manufacturer's protocol. No co-localization with EGFP signal was shown, indicating that BoHV-4EGFPΔTK did not infect neurons (Fig 3a, 3b, 3c). For immunohistochemistry, sections were rinsed with PBS, permeabilized with 1% Triton-X 100 in PBS for 10 min, blocked with bovine serum albumin (0.4% in PBS), incubated overnight with the astrocyte marker anti-GFAP antibody (Sigma, diluted 1:250 in PBS) in a humid chamber. After rinsing the sections, sections were incubated with secondary antibody (antimouse Alexa 568, Molecular Probes, Eugene, OR, 1:250 in PBS) for 3 hours at 4°C in humid chamber. Some of the EGFP expressing cells in the RMS area were labelled with the anti-GFAP signal and were identified as astrocyte cells (Fig 4a, 4b, 4c).

The most interesting observation made during this study, was the ability of BoHV-4EGFPΔTK to replicate in highly replicating glioma cells but not in post mitotic brain cells. This observation could be explained by a proteomic switch occurring in the intracellular microenvironment of tumor cells capable of activating the full replication cycle of BoHV-4EGFPΔTK.

The absence of pathogenicity in the rat brain and the ability to establish a permissive infection in cultures of glioma cells, make BoHV-4 an ideal candidate as a gene delivery or oncolytic vector for gliomas in the nervous system.

## Authors' contributions

Redaelli M., carried out the in vivo and in vitro experiments and helped to draft the manuscript. Cavaggioni A., participated in the design of the study and helped to draft the manuscript. Mucignat-Caretta C., participated in the design of the study, analyzed the frozen tissue section and helped to draft the manuscript. Caretta A., participated in the design of the study and prepared the frozen tissue section. Donofrio G., designed the study, prepared the viral vector and helped to draft the manuscript. All authors read and approved the final manuscript.
